# Sex-Based Mechanisms of Stress-Induced Alcohol-Seeking

**DOI:** 10.3390/bs16020311

**Published:** 2026-02-23

**Authors:** Anna C. S. Garrison, Evgeny Jenya Chumin, Mario Dzemidzic, Julia L. Smith, Wei Wu, Ann E. K. Kosobud, David A. Kareken, Sean O’Connor, Martin H. Plawecki, Melissa A. Cyders

**Affiliations:** 1 Department of Psychology, Indiana University Indianapolis, Indianapolis, IN 46202, USA; 2 Center for Neuroimaging, Medical Imaging Research Institute, Department of Radiology & Imaging Sciences, Indiana University School of Medicine, Indianapolis, IN 46202, USA; 3 Indiana Alzheimer’s Disease Research Center, Indiana University School of Medicine, Indianapolis, IN 46202, USA; 4 Department of Neurology, Indiana University School of Medicine, Indianapolis, IN 46202, USA; 5 Department of Psychiatry, Indiana University School of Medicine, Indianapolis, IN 46202, USA

**Keywords:** intravenous alcohol, alcohol administration, subjective alcohol response, resting-state connectivity, depression, anxiety, low-level response to alcohol

## Abstract

Sex differences in stress-induced alcohol-seeking are well documented. The overarching goal of this study is to examine how sex may moderate the relationship between depression and anxiety symptoms and stress-induced alcohol-seeking and to identify mechanisms of this relationship. We explore subjective alcohol responses and the resting-state functional connectivity of the amygdala and the hippocampus, regions implicated in anxiety and depression, as potential sex-dependent mediators. This secondary analysis draws from a recently published trial of 84 adults aged 21 to 55 (54.8% female, mean age = 32, SD = 10.68; 58.3% White, 88.1% non-Hispanic/Latino) who endorsed moderate-to-heavy alcohol use. All participants completed two counterbalanced intravenous alcohol administration sessions, and 54 completed optional neuroimaging. Generalized anxiety symptoms were significantly associated with greater stress-induced alcohol-seeking in women but not in men. Depression symptoms showed a similar pattern, though the results did not reach statistical significance. Across men and women, blunted state stimulation, but not state anxiety, in response to alcohol was associated with greater stress-induced alcohol-seeking. In men, anxiety symptoms were linked with heightened state stimulation effects, which appeared to buffer against stress-induced alcohol-seeking. State stimulation findings may suggest a possible mechanism for sex differences concerning anxiety pathways to alcohol-seeking. Subjective alcohol responses did not mediate the relationship between depression symptoms and stress-induced alcohol-seeking. Resting-state network connectivity findings identified several potential sex-dependent neural mechanisms that warrant further investigation. Although this study was not originally designed as a direct test of competing subjective response and low-level response to alcohol theoretical models, our findings are consistent with Schuckit’s low level of response to alcohol theory. Our findings showed that blunted stimulation may contribute to stress-induced alcohol-seeking among men. Identifying mechanisms that underlie sex-specific relationships with stress-induced alcohol-seeking can inform the development of tailored intervention approaches, ultimately enhancing treatment efficacy for both men and women.

## 1. Introduction

Stress contributes to the development and maintenance of alcohol-seeking behaviors in both human (Brady & Sonne, 1999; K. Keyes et al., 2012) and animal (Domi et al., 2021) models. Women experience higher rates of anxiety (Jalnapurkar et al., 2018) and depression (Albert, 2015) and are more likely to drink in response to acute stress (Guinle & Sinha, 2020; Patock-Peckham et al., 2022; Peltier et al., 2019), contributing to faster progression to alcohol use disorder (AUD) (K. M. Keyes et al., 2010). Depressive symptoms are more strongly linked to alcohol use in women (Jeong et al., 2019; Karpyak et al., 2019; Moscato et al., 1997; Zhan et al., 2012), whereas findings for anxiety are mixed: some studies report stronger anxiety–alcohol associations in women (Johannessen et al., 2017; Kendler et al., 2015), while others find stronger effects in men (Atkinson & Finn, 2019; Schry et al., 2014; Torres et al., 2023; Zale et al., 2019). Overall, evidence supports sex-specific links between stress and alcohol use, but underlying mechanisms remain unclear.

Subjective responses, i.e., individual differences in positive (e.g., stimulation and euphoria) and negative (e.g., sedation and dysphoria) alcohol effects (A. King et al., 2021), are one candidate mechanism, as they predict AUD risk (Morean & Corbin, 2010; Quinn & Fromme, 2011). A key positive subjective response, stimulation, captures increases in state energy or excitement after consuming alcohol, which predicts greater alcohol craving, intake, and AUD risk (Bujarski & Ray, 2014; Fischer et al., 2023; Green et al., 2019; A. King et al., 2021; Ray et al., 2010). A key negative subjective response, anxiety, captures reductions in state anxiety after consuming alcohol (Wilson, 1982). Alcohol produces anxiolytic effects (i.e., reduces anxiety, tension, or nervousness) (Bradford et al., 2017; Hendler et al., 2011) that are stronger in those with high trait anxiety (Stewart & Pihl, 1994), which predict craving (Cheng et al., 2022; McCaul et al., 2017) and AUD risk (Schmidt et al., 2007; Smith & Randall, 2012). Many anxious individuals drink to relieve anxiety (Book & Randall, 2002; McCaul et al., 2017), increasing risk for problematic use (Anker & Kushner, 2019).

The resting-state network connectivity of the amygdala and hippocampus, regions central to emotion and stress regulation, is another candidate mechanism. Resting-state functional connectivity refers to the temporal correlation of brain activity between brain regions measured while a participant is not engaged in an explicit task. Altered amygdala connectivity and reduced hippocampal connectivity are implicated in anxiety and depression (Gold et al., 2017; Jacob et al., 2022; Langhammer et al., 2025; Tang et al., 2018). The connectivity of these regions is also linked to alcohol use: stronger amygdala–orbitofrontal connectivity predicts alcohol use (Crane et al., 2018; Peters et al., 2017), reduced amygdala connectivity with the dorsal anterior cingulate cortex and caudate nucleus relates to alcohol misuse and dependence (Hu et al., 2018; Sun et al., 2024), and reduced hippocampal–frontal connectivity relates to craving and problematic use (Arienzo et al., 2020).

Importantly, both subjective responses and resting-state connectivity show sex effects, positioning them as strong candidates for explaining sex differences in stress-induced alcohol-seeking. Men often report stronger stimulation effects than women, which predict drinking escalation (A. C. King et al., 2011; Mumenthaler et al., 1999), though findings are mixed (see Quinn & Fromme, 2011). Animal studies suggest that females show greater alcohol-related anxiety reduction than males (D. B. Matthews & Kerr, 2024); this is untested in humans. In neural circuits, women show greater amygdala reactivity and altered amygdala–prefrontal connectivity in depression (Almeida et al., 2011; Lebron-Milad et al., 2012), whereas men show stronger associations between alcohol misuse and reduced amygdala–dorsal anterior cingulate connectivity (Claus et al., 2011; Hu et al., 2018).

The overarching goal of this study is to examine how sex may moderate the relationship between depression and anxiety symptoms and stress-induced alcohol-seeking. It is our secondary goal to test subjective responses and resting-state connectivity as mechanisms explaining these differences. The present study integrates behavioral, subjective, and neural measures in a controlled experimental framework. Self-report of alcohol use can be biased by sex differences in social desirability and disclosure willingness (Boniface et al., 2014; Davis et al., 2010). Using data from a recently published intravenous (IV) alcohol self-administration trial (Garrison et al., 2025), this study employs objective measures of alcohol-seeking. IV administration eliminates expectancy effects and standardizes alcohol exposure (Cyders et al., 2020), reducing pharmacokinetic sex differences and allowing for precise assessment of alcohol-seeking and subjective response. Resting-state functional connectivity provides insight into the stable neural network architecture underlying mood symptoms.

First, we tested whether sex moderates the relationship between anxiety and depression symptoms and stress-induced alcohol-seeking. We hypothesized that women would show stronger positive associations between anxiety and depression symptoms and alcohol-seeking compared to men.

Second, we examined state stimulation and state anxiety subjective responses to alcohol as mediators of these sex-specific relationships. We hypothesized that changes in subjective responses would mediate the relationship between anxiety and depression symptoms and stress-induced alcohol-seeking. We also hypothesized that sex would moderate both the symptom-to-state subjective response and symptom-to-alcohol-seeking paths, such that higher symptoms would predict greater state stimulation and lower state anxiety and that the relationships would be stronger in women compared to men.

Finally, we investigated sex differences in resting-state network connectivity of the amygdala and hippocampus with anxiety and depression symptoms and stress-induced alcohol-seeking. We hypothesized that sex would moderate the relationship between anxiety and depression symptoms and amygdala/hippocampal connectivity with cortical resting-state brain networks. We also hypothesized that sex would moderate relationships between amygdala/hippocampal connectivity and stress-induced alcohol-seeking.

## 2. Materials and Methods

### 2.1. Participants

Data were collected from 84 healthy, community-dwelling adults aged 21–55 (54.8% women) recruited via advertisements. Participants were required to currently use alcohol and were excluded if pregnant or breastfeeding; seeking treatment for substance use; legally prohibited from drinking; or they had medical, psychiatric, or medication-related contraindications that would compromise data quality or participant safety. Individuals showing signs of intoxication at any visit (via urinalysis, breath alcohol measurement, field sobriety test) were also excluded (see Garrison et al. (2025), for full details).

### 2.2. Measures

Demographic information was collected via open-ended interview questions, including age, sex assigned at birth, race, ethnicity, and education level.

Anxiety Symptoms were assessed via the Generalized Anxiety Disorder 7 (GAD-7) questionnaire (Spitzer et al., 2006). Participants rated the frequency of seven anxiety symptoms (e.g., feeling nervous, anxious, or on edge; not being able to stop or control worrying; trouble relaxing; feeling afraid as if something awful might happen) over the previous two weeks, on a scale from 0 (not at all) to 3 (nearly every day). Total scores range from 0 to 21. The GAD-7 has excellent internal consistency (Cronbach’s alpha = 0.92) and strong construct and convergent validity (Spitzer et al., 2006).

Depression Symptoms were assessed via the Center for Epidemiologic Studies Depression Scale (CES-D) (Radloff, 1977). Participants rated the frequency of 20 depression symptoms (e.g., I felt sad; I thought my life had been a failure; I did not feel like eating; I could not get “going”) over the previous week, on a scale from 0 (rarely or none of the time: less than 1 day) to 3 (most or all of the time: 5–7 days). Total scores range from 0 to 60. The CES-D has high internal consistency (Cronbach’s alpha = 0.90) and validity in adult samples (Cosco et al., 2017).

State subjective alcohol responses were assessed via nine self-report questions administered throughout the alcohol infusion session, in a manner consistent with our prior work (Plawecki et al., 2022). Each item was rated on a 0–100 scale. The current analysis only used two of these items: “How ANXIOUS (tense, jittery, nervous) do I feel right now?” which we label as “state anxiety,” and “How STIMULATED (lively, energized and excited) do I feel right now?” which we label as “state stimulation.”

Recent drinking history was assessed using the 35-day Timeline Follow-Back (TLFB; Sobell & Sobell, 1992). Participants reviewed a calendar and reported the number of standard drinks consumed each day, using life events as memory anchors. Variables derived included drinking days per week, drinks per week, and drinks per drinking day. The TLFB demonstrates high test–retest reliability and validity (Sobell & Sobell, 1992).

### 2.3. Procedures

See Garrison et al. (2025) for full details. After screening and interviews, participants completed two counterbalanced IV alcohol sessions: one with neutral audiovisual stimuli and one with aversive audiovisual stimuli. IV alcohol was delivered using the Computer-Assisted Alcohol Infusion System (CAIS) software, which standardized alcohol exposure across participants and prevented levels from exceeding 180 mg/dL (Zimmermann et al., 2009; Zimmermann et al., 2008). Each session began with a 40 min alcohol priming interval: participants’ breath alcohol concentrations (BrACs) were raised to 60 mg/dL over 15 min and maintained there for ~25 min (Kosobud et al., 2015; O’connor et al., 1998; Plawecki et al., 2018; Ramchandani et al., 1999; Ramchandani et al., 2002). Subjective responses were collected at baseline (0 mg/dL), at the start of the prime (60 mg/dL), and at the end of the maintenance period. Participants then completed a 2.5 h progressive-ratio alcohol self-administration task in which they could choose to work for alcohol or saline (“water”) rewards. Alcohol rewards increased BrAC by 10 mg/dL over 2.5 min, followed by a decline of −0.8 mg/dL/min. Work consisted of Constant Attention Task (CAT) trials (Plawecki et al., 2013), with an escalating number of successful trials required to earn each subsequent reward and difficulty adjusted in real time to maintain ~50% success. Participants remained on site until 7 p.m. or until BrAC dropped below 35 mg/dL, after which participants were dismissed and allowed to travel home however they wished.

A subset of 54 participants completed an optional fMRI session at least seven days later. Eligible participants were right-handed and free of imaging contraindications. Imaging was conducted on a Siemens 3T Prisma (Erlangen, Germany) with a 64-channel head coil array. High-resolution anatomical volume (3D Magnetization Prepared RApid Gradient Echo sequence (MPRAGE), 0.8 × 0.8 × 0.8 mm^3^ voxels) followed Human Connectome Protocol parameters (detailed in our prior work: Fisher-Fox et al., 2025). Resting-state BOLD data consisted of 616 whole-brain volumes using a multiband echo-planar imaging (EPI) sequence (Center for Magnetic Resonance Research at the University of Minnesota, gradient echo, repetition/echo time (TR/TE) = 780/29 ms, flip angle 54 deg, field-of-view 220 × 220 mm^2^, matrix 88 × 88, fifty-five 2.5-mm thick slices, 2.5 × 2.5 × 2.5 mm^3^ voxel, slice acceleration factor = 5) (as detailed in our prior work: Fisher-Fox et al., 2025), preceded by paired phase-reversed spin echo field maps (3 A-P and 3 P-A phase direction volumes, TR/TE = 1200/64.40 ms).

Structural and functional preprocessing used an in-house Bash/Python 3.6 pipeline with the FMRIB Software library (FSL version 6.0.1) (Eklund et al., 2016; Halcomb et al., 2024). Structural volumes were denoised (Coupe et al., 2008), bias field-corrected, tissue-type-segmented (FSL fslanat), and skull-stripped (ANTs) (Avants et al., 2011). Montreal Neurological Institute (MNI) space-to-structural-volume transformation (FSL flirt and fnirt) was estimated and applied to register the Schaefer 200-cortical (Schaefer et al., 2018) brain region and Melbourne Subcortical Atlas Scale II (Tian et al., 2020) parcellations to each participant’s anatomical space. Native space BOLD data were preprocessed (as described in Fisher-Fox et al., 2025): distortion correction (topup/applytopup), head motion realignment (mcflirt), linear and nonlinear registration to MNI space, normalization to mode 1000, and spatial smoothing with a 6 mm isotropic full width at half maximum Gaussian kernel, independent component analysis (ICA)-based decomposition (via melodic), and ICA-AROMA denoising (Pruim et al., 2015). A single-step nuisance regression (Phạm et al., 2023) included motion parameters (with derivatives and squares), aCompCor physiological components (Muschelli et al., 2014), high-pass filtering (*f_min_* = 0.009 Hz) (Shirer et al., 2015), and DVARS-based outlier volume correction (Power et al., 2014). On average, 1.28% of volumes (SD = 1.23%; range = 0–7.29%) were flagged as outliers.

### 2.4. Data Analysis Plan

Data were compiled, cleaned, and checked for missingness, normality, sample characteristics, and correlations. Independent samples *t* tests evaluated sex differences. First, we conducted simple moderation analyses in SPSS 31.0.0.0 using Process Model 1 (Hayes, 2017). Mean anxiety or depression symptoms were independent variables in separate models; total trials completed for alcohol in the aversive session were the dependent variable. Sex (women = 0, men = 1) was the moderator, and the total trials completed for alcohol in the neutral session were a covariate to isolate stress-induced alcohol-seeking.

Second, we determined the appropriate change model for each subjective alcohol response using growth curve models (GCMs) in MPlus 8.11 (Muthén & Muthén, 2017) across baseline, prime start, and prime end. Linear and nonlinear latent-basis GCMs (Chou et al., 1998; Meredith & Tisak, 1990) were compared (Table 1). For state anxiety, the linear and nonlinear models fit the data equally well; we adopted the linear model for simplicity. For state stimulation, the nonlinear model fit best and was adopted, including two parameters: level (baseline) and shape (nonlinear change). We then constructed moderated mediation models by combining the models testing whether sex moderates the relationship between anxiety and depression symptoms on stress-induced alcohol-seeking with the GCMs. The linear slope factor (state anxiety) or nonlinear shape factor (state stimulation) was a mediator, and sex was a moderator of the effects of depression or anxiety symptoms.

Third, we examined asymmetric, cortico-subcortical functional connectivity (FC). FC refers to the temporal correlation of signal fluctuations between spatially distinct brain regions, indicating coordinated neural activity. FC was calculated as the Pearson correlation between the mean time series of four medial temporal lobe regions (bilateral anterior/posterior hippocampus and medial/lateral amygdala) and the 200 Schaefer cortical parcels (Schaefer et al., 2018), yielding an 8 × 200 FC matrix per participant. Brain regions were grouped into four subcortical (left/right hippocampus and amygdala) and 7 cortical (Yeo et al., 2011) resting-state networks for network (block)-level analysis (Chumin et al., 2024; Sripada et al., 2014). Network contingent analysis (NCA) was used to perform block-level inference and address mass univariate testing. For each of the 8 × 200 edges, we (1) fit linear regression models (MATLAB 2023b fitlm) to obtain *t*-values; (2) generated 5000 permutation-based null *t*-value distributions by shuffling the predictor; (3) computed permutation *p*-values as the proportion of null t-values exceeding the observed absolute *t*-value; and (4) compared the number of significant edges (permutation *p* < 0.05) within each block to a permutation-derived null distribution of edge counts to obtain block-level *p*-values. False discovery rate (FDR) correction (*p* < 0.05 across 28 blocks) determined final block-level significance. Three linear regression models were tested using NCA: (1) the relationship between anxiety symptoms (GAD-7, predictor) and FC, (2) the relationship between depression symptoms (CES-D, predictor) and FC, and (3) the relationship between FC (predictor) and trials completed for alcohol in the aversive session. Age was a covariate in all models. Alcohol-seeking in the neutral session was a covariate to isolate stress-induced alcohol-seeking. Each model evaluated the main effects of the predictor and sex and their interaction.

## 3. Results

### 3.1. Preliminary Analyses

The sample included 84 adults (mean age = 32, SD = 10.68; 58.3% White, 88.1% non-Hispanic/Latino), 54 of whom contributed usable imaging data (Supplemental Table S1). Study variables distributions were approximately normal (skewness < 2, kurtosis < 7). There were no significant sex differences in depression or anxiety symptoms or in alcohol-seeking (*p*s > 0.05). Women reported higher baseline state anxiety and stimulation (*p*s = 0.03–0.05; Table 2), though effect sizes were small (Cohen’s *d* < 0.5). No significant sex differences remained at prime initiation or end (*p*s = 0.14–0.91; Table 2), even though aversiveness of stimuli was maintained throughout the prime (see Garrison et al., 2025). Table 3 provides the correlations by sex (see Supplemental Table S2 for descriptions and correlations).

### 3.2. Tests of Sex Moderation for Anxiety and Depression Symptoms

**Figure 1:**
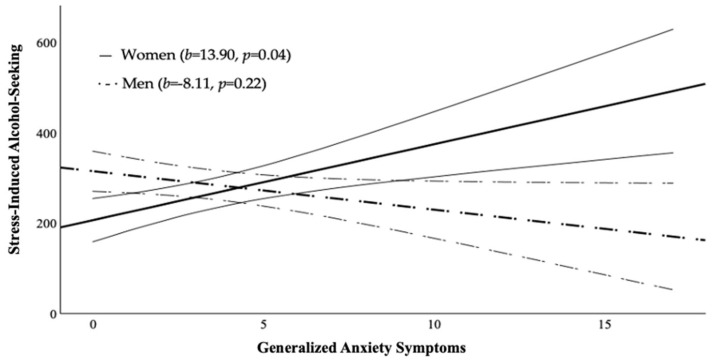
Relationship between generalized anxiety symptoms and stress-induced alcohol-seeking for women and men. Note: Bold solid line represents women, bold dashed line represents men. Curved lines represent 95% Confidence Intervals.

**Figure 2:**
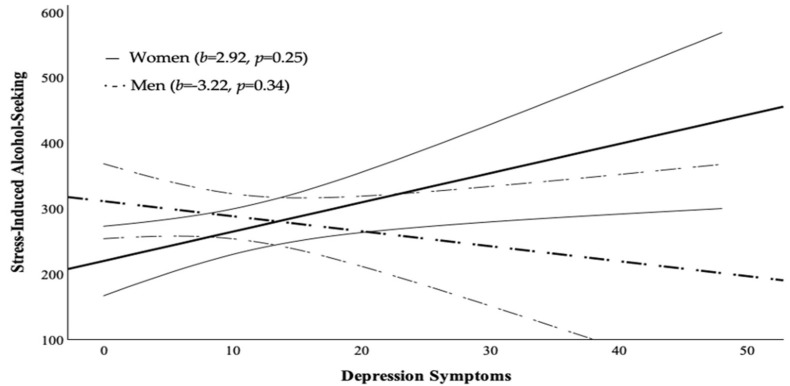
Relationship between depression symptoms and stress-Induced alcohol-seeking for women and men. Note: Bold solid line represents women, bold dashed line represents men. Curved lines represent 95% Confidence Intervals.

The hypothesis that sex would moderate the relationship between anxiety symptoms and stress-induced alcohol-seeking was supported (Table 4): generalized anxiety symptoms and sex significantly interacted (*b* = −22.01, *p* = 0.02). Simple slope analysis showed a significant positive relationship between anxiety symptoms and stress-induced alcohol-seeking for women (*b* = 13.90, *p* = 0.04) and a non-significant negative association for men (*b* = −8.11, *p* = 0.22; Figure 1). The hypothesis that sex would moderate the relationship between depression symptoms and stress-induced alcohol-seeking was not supported (Table 5), although the pattern resembled that observed for anxiety symptoms (Figure 2).

### 3.3. Tests of Moderated Mediation Using Change in Subjective Alcohol Response

The hypothesis that state anxiety change would mediate the sex-specific relationship between generalized anxiety symptoms and stress-induced alcohol-seeking was partially supported (Table 6): generalized anxiety symptoms were positively associated with stress-induced alcohol-seeking (*b* = 0.16, *p* = 0.01), and there was a significant sex difference in stress-induced alcohol-seeking (*b* = 1.03, *p* = 0.04). Sex significantly moderated the relationship between anxiety symptoms and stress-induced alcohol-seeking (*b* = −0.24, *p* = 0.01): women showed a significant positive relationship (*b* = 0.16, *p* = 0.01), whereas men showed a non-significant negative relationship (*b* = −0.08, *p* = 0.25). Sex did not moderate the relationship between anxiety symptoms and state anxiety change, nor were anxiety symptoms related to state anxiety change. State anxiety change was not significantly associated with stress-induced alcohol-seeking (*b* = −8.36, *p* = 0.08).

Baseline state anxiety was negatively related to stress-induced alcohol-seeking (*b* = −3.06, *p* < 0.01), such that lower baseline state anxiety was associated with more stress-induced alcohol-seeking. We conducted an exploratory analysis with baseline state anxiety as mediator (Table 7). The relationship between anxiety symptoms and baseline state anxiety fell just short of significance (*b* = 2.00, *p* = 0.051); sex did not significantly moderate the relationship. Lower baseline state anxiety (*b* = −1.47, *p* < 0.01) and higher anxiety symptoms (*b* = 16.83, *p* = 0.02) were positively associated with more stress-induced alcohol-seeking. Sex moderated the relationship between baseline state anxiety and stress-induced alcohol-seeking.

**Figure 3:**
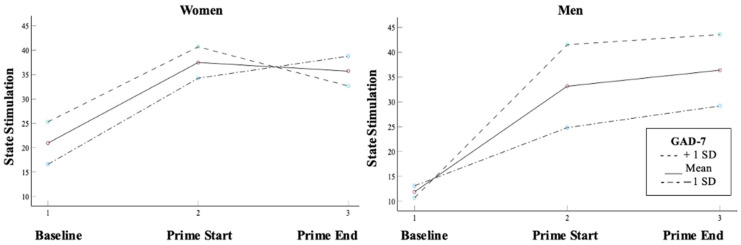
State stimulation change trends across men and women at three levels of anxiety symptoms. Note: GAD-7 = Generalized Anxiety Disorder-7 score. +1 SD indicates estimated change in state stimulation for participants one standard deviation above the mean GAD-7 score; mean indicates estimated change in state stimulation for participants at the mean GAD-7 score; −1 SD indicates estimated change in state stimulation for participants one standard deviation below the mean GAD-7 score.

Sex significantly moderated the relationship between anxiety symptoms and state stimulation change (*b* = 0.03, *p* = 0.02): women showed a non-significant relationship (*b* = −0.01, *p* = 0.25), whereas men showed a significant positive relationship (*b* = 0.02, *p* = 0.03) (Table 8; Figure 3). The relationship between the state stimulation changes and stress-induced alcohol-seeking was negative and not significant (*b* = −1.56, *p* = 0.07). Sex moderated the relationship between the state stimulation changes and stress-induced alcohol-seeking. The hypothesis that changes in state anxiety and state stimulation would mediate the sex-specific relationship between depression and stress-induced alcohol-seeking was not supported (Supplemental Tables S3 and S4).

### 3.4. Tests of Sex Differences in Resting-State Network Connectivity

**Figure 4:**
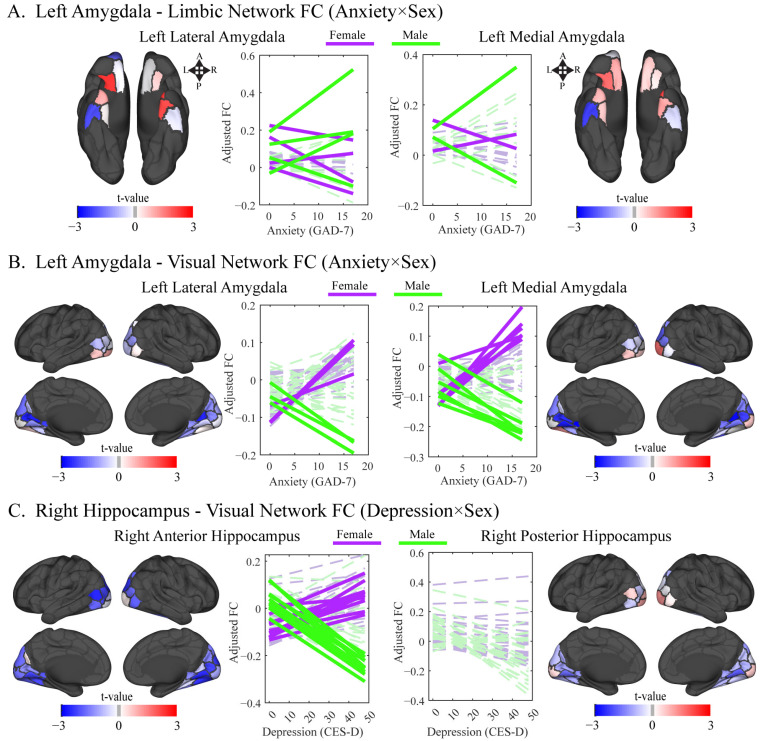
Sex interaction with anxiety and depression as predictors of functional connectivity (FC). Note: Cortical surface visualizations of empirical *t*-values for significant anxiety × sex (**A**,**B**) and depression × sex (**C**) interactions from linear model testing. Cortico-subcortical block-level significance was set at *p_FDR_* < 0.05, with FC edge t-values projected onto the cortical Yeo et. al. (2021), resting-state network regions for all functional connections of that significant block. Line plots (colored by sex) illustrate edge-level interactions (amygdala to cortical region) within each significant FC block, with solid lines representing edges that contributed to block-level significance (permutation *p* < 0.05; see data analysis plan for details). Generalized Anxiety Disorder 7 (GAD-7); Center for Epidemiologic Studies Depression Scale (CES-D).

The hypothesis that sex would moderate the relationship between anxiety symptoms and connectivity was partially supported. There were no FDR-corrected significant effects of anxiety symptoms or sex. A significant anxiety by sex interaction emerged in FC blocks linking the left amygdala with limbic and visual cortical resting-state networks (*p_FDR_* < 0.05). In the left amygdala–limbic block, edges varied in direction (Figure 4A), whereas in the left amygdala–visual block, women showed positive and men negative associations (Figure 4B).

The hypothesis that sex would moderate the relationship between depression symptoms and connectivity was supported. Significant main effects of depression symptoms were found for FC blocks linking the left amygdala with the default mode and somatomotor networks (*p_FDR_* < 0.05). A main effect of sex was observed for the right hippocampus-visual network block, which was also the only block where a significant depression by sex interaction emerged (*p_FDR_* < 0.05). In this block, contributing edges showed consistent positive associations in women and negative associations in men (Figure 4C).

**Figure 5:**
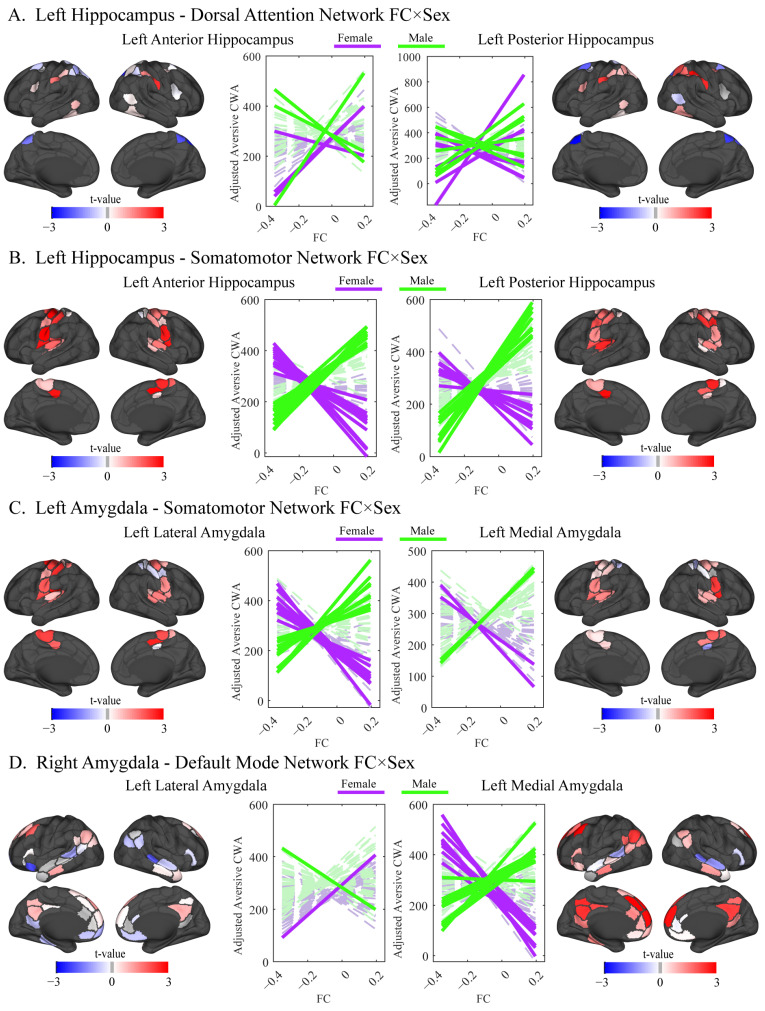
Functional connectivity (FC) by sex interaction as predictors of stress-induced alcohol-seeking. Note: Cortical surface visualizations of empirical t-values for significant FC × sex and interactions from linear model testing, where FC was a predictor of alcohol-seeking in the aversive condition. Cortico-subcortical block-level significance was set at *p_FDR_* < 0.05, with FC edge *t*-values projected onto the cortical Yeo et al. (2011), resting-state network regions for all functional connections of that significant block. Line plots (colored by sex) illustrate edge-level interactions within each significant FC block, with solid lines representing edges that contributed to block-level significance (permutation *p* < 0.05; see data analysis plan for details).

The hypothesis that sex would moderate the relationship between connectivity and stress-induced alcohol-seeking was supported. Significant main effects emerged for FC between the hippocampus (left and right) and amygdala (right only) with the default mode network, as well as for FC between the left hippocampus and the dorsal attention network, and between the left hippocampus/right amygdala and the visual network (all *p_FDR_* < 0.05). No main effects of sex emerged. Among blocks with significant main effects, FC by sex interactions emerged for the left hippocampus–dorsal attention network and the right amygdala–default mode network (*p_FDR_* < 0.05). In the left hippocampus–dorsal attention network block, edges showed opposing directions for men and women (Figure 5A). In the left amygdala–default mode network block, significance was driven by the left medial amygdala, which showed a consistent negative association in women and a weak positive association in men (Figure 5D). Additional FC by sex interactions were found for the left hippocampus and amygdala FC with the somatomotor network, with all contributing edges showing negative associations with stress-induced alcohol-seeking in women and positive associations in men (*p_FDR_* < 0.05; Figure 5B,C).

## 4. Conclusions

This study provides new mechanistic insight into how stress influences alcohol-seeking behavior in men and women. Using an objective model of stress-induced alcohol-seeking paired with subjective response and resting-state connectivity measures, we demonstrate sex-specific pathways linking anxiety, alcohol response, and neural circuitry to drinking behavior. Unlike prior work relying primarily on retrospective self-report, this study captured real-time alcohol-seeking in response to stress and revealed distinct pathways through which anxiety and depression symptoms may influence alcohol use, including both alcohol subjective responses and cortico-limbic networks. These findings highlight the importance of examining multiple levels of analysis—behavioral, subjective, and neural—to better characterize risk processes that differ for women and men.

As hypothesized, we found stronger associations between anxiety symptoms and stress-induced alcohol-seeking among women, which aligns with previous work showing that women are more likely to drink alcohol in response to anxiety (Johannessen et al., 2017; Kendler et al., 2015; McCaul et al., 2019) but conflicts with previous work linking anxiety to alcohol problems and consumption in men (Atkinson & Finn, 2019; Ebbert et al., 2018; Schry et al., 2014; Torres et al., 2023; Zale et al., 2019). One important explanation for these discrepant findings is differences in how anxiety has been operationalized across studies. The present study assessed generalized anxiety symptoms, whereas several prior studies, most notably Ebbert et al. (2018), examined anxiety sensitivity, a related but distinct construct reflecting concern about the social or physical consequences of anxiety-related sensations. These constructs have been shown to exhibit different associations with alcohol-related outcomes, including sex-specific patterns. Methodological variation in alcohol-use assessment and stress context may further contribute to discrepancies in the literature. Many prior studies have relied on self-reported alcohol use over extended periods, which can reflect averaged behavior and may be subject to recall bias, whereas the current study employed an objective measure of alcohol-seeking that directly assessed behavior in response to specific, aversive circumstances. Moreover, the present study examined alcohol-seeking following acute stress, which may differ from drinking associated with chronic stress exposure. Consistent with this distinction, prior research suggests that men may be more physiologically affected by chronic stress (K. A. Matthews et al., 2001), whereas women demonstrate greater behavioral (Patock-Peckham et al., 2022) and neural responses to acute stress (Grodin et al., 2024). Overall, these findings suggest that generalized anxiety symptoms may represent particularly relevant prevention and intervention targets for reducing alcohol misuse in women, but not necessarily in men.

Contrary to our hypotheses, depression symptoms related to stress-induced alcohol-seeking similarly across men and women. This was unexpected, given prior research indicating that depression symptoms are more strongly associated with alcohol use among women than men (Jeong et al., 2019; Karpyak et al., 2019; Moscato et al., 1997; Zhan et al., 2012). However, the directions of the observed relationships align with previous research and hypotheses: a positive relationship was found in women, while no relationship was observed in men. This pattern may indicate small sex differences that could emerge in a larger, statistically better-powered study.

Contrary to our hypotheses and previous findings (D. B. Matthews & Kerr, 2024; Stewart & Pihl, 1994), neither anxiety symptoms nor sex was related to state anxiety change across the alcohol priming interval. Such inconsistency may reflect differences in how anxiety was measured, such as anxiety sensitivity, state anxiety, trait anxiety, and anxiety disorder symptoms. In the current work, baseline state anxiety and generalized anxiety symptoms were both significantly related to stress-induced alcohol-seeking, but in opposite directions: higher anxiety symptoms were associated with more stress-induced alcohol-seeking, whereas higher state anxiety before alcohol administration was associated with less stress-induced alcohol-seeking. These diverging relationships underscore the importance of measuring both anxiety symptoms (e.g., over days, weeks, or months) and idiosyncratic, momentary experiences of anxiety, as their effects may differ and capture distinct processes to help explain mixed findings in the literature (Atkinson & Finn, 2019; Johannessen et al., 2017; Kendler et al., 2015; Schry et al., 2014; Torres et al., 2023; Zale et al., 2019). Anxiety symptoms may reflect broader difficulties regulating state anxiety that increase risk for drinking, whereas acute state anxiety in the laboratory may temporarily suppress alcohol-seeking behavior. Although the novelty of the IV alcohol procedure could contribute to this effect, counterbalancing reduces this concern. While increasing momentary anxiety is not a viable intervention target, state anxiety may nonetheless help identify individuals who respond differently to stress in drinking contexts.

The state stimulation findings suggest a possible mechanism for sex differences: anxiety symptoms related to higher state stimulation increase among men, which in turn is related to less stress-induced alcohol-seeking. This pattern is consistent with theories linking low alcohol sensitivity to greater AUD risk (Kalu et al., 2012; Schuckit, 1994; Schuckit et al., 1997), though evidence is mixed (A. C. King et al., 2014, 2016, 2019; A. King et al., 2021). Our findings extend this work by suggesting that blunted stimulation may also contribute to stress-induced alcohol use. Mediation effects for depression symptoms were small and not significant, suggesting that state anxiety and state stimulation are not meaningful mediators for the relationship between depression symptoms and stress-induced alcohol-seeking. These results highlight the need to further explore alternative mechanisms of the relationship between depression and stress-induced alcohol use, including its moderation by sex.

We identified several sex-dependent resting-state connectivity patterns involving the amygdala and hippocampal networks. For generalized anxiety symptoms, sex differences emerged in connectivity between the left amygdala and the limbic and visual networks, consistent with prior evidence of limbic dysregulation and sex-dependent amygdala lateralization (He et al., 2016; Kilpatrick et al., 2006; Ocklenburg et al., 2022). For depression, sex-dependent effects involved the right hippocampus and the visual network, aligning with work implicating hippocampal circuitry (Posener et al., 2003; Sheline, 2011) and visual networks (Ocklenburg et al., 2022) in depression. For stress-induced alcohol-seeking, sex-dependent associations appeared in networks involving dorsal attention, default mode, and somatomotor systems, networks previously implicated in alcohol cue reactivity, AUD-related disconnectivity, and structural abnormalities (Chumin et al., 2019; Radoman et al., 2024; Rice et al., 2024; Seo et al., 2011; Song et al., 2021).

Although this study was not designed as a direct test of competing theoretical models, our findings speak to ongoing debates between subjective response frameworks, including Schuckit’s low level of response to alcohol theory (Kalu et al., 2012; Schuckit, 1994; Schuckit et al., 1997). The association between blunted stimulation and greater stress-induced alcohol-seeking may align with low-response accounts, suggesting that reduced positive alcohol effects increase risk for heavier use. In contrast, a lack of findings with anxiety suggests that stimulation-based pathways may play a more prominent role in this acute stress context. Rather than positioning these perspectives as mutually exclusive, our results suggest they may reflect complementary mechanisms that operate under different conditions or in different individuals. Future work explicitly comparing these models, particularly using controlled alcohol administration paradigms, will clarify when stimulation, changes in anxiety, or other subjective responses most strongly confer risk.

The current work has several implications. First, the relationships found here highlight the importance of studying sex-specific mechanisms in AUD research (Guinle & Sinha, 2020; Patock-Peckham et al., 2022; Peltier et al., 2019), using objective behavioral assessments, including alcohol administration. Further research is needed to determine whether stimulation effects represent a safe and effective mechanism to reduce alcohol-related risk in men. Women may benefit more from targeting generalized anxiety symptoms directly to reduce stress-induced alcohol-seeking. Second, anxiolytic and stimulation effects are not likely to be mechanisms linking depression symptoms with stress-induced alcohol-seeking. Third, the resting-state findings implicate the amygdala and hippocampus in sex-dependent alcohol behavior, and they could serve as promising targets for future neuroimaging studies aimed at identifying biomarkers of risk, resilience, and potentially circuit-level interventions with techniques such as repetitive transcranial magnetic stimulation. Finally, the divergence between anxiety at the symptom-level and momentary state anxiety response underscores the importance of multi-level measurement approaches to better capture dynamic risk processes.

These findings should be considered in light of several limitations. First, the modest sample size may have limited power. Second, because the study recruited healthy, heavy-drinking adults for safety during alcohol infusion and imaging, the results may not generalize to lighter drinkers or individuals across the full AUD spectrum, despite evidence that coping-related drinking and its sex differences occur broadly (Corbin et al., 2013; Foster et al., 2014; Gilson et al., 2017; Holahan et al., 2001; Park & Levenson, 2002; Rodriguez et al., 2020). Similarly, we intentionally balanced the sex ratio to maximize power for detecting sex effects. Therefore, our sample does not reflect population AUD prevalence, although the gender gap is narrowing (Agabio et al., 2017; K. M. Keyes et al., 2011; Verplaetse et al., 2025). Third, these findings may not extend to descending-limb alcohol effects or other alcohol-seeking contexts. Fourth, while IV alcohol administration reduces self-report bias and tightly controls exposure (Cyders et al., 2020), alcohol is typically consumed orally and in the evening, and IV administration may have influenced baseline state anxiety; complementary oral-administration studies during evening hours are needed. Fifth, drinking history was not included as a covariate (due to limited sample size, the lack of significant sex differences in drinking history in this sample, and IV alcohol’s ability to ensure equal alcohol exposure within and across participants) and should be examined in future work. Finally, limited power precluded integrating resting-state connectivity into moderated mediation models; these findings should guide larger mechanistic studies.

These findings highlight the value of integrating sex-specific approaches into AUD research, particularly using objective alcohol administration paradigms. Subjective alcohol responses and amygdala and hippocampal connectivity emerged as promising pathways that differ by sex. Future work should determine the clinical utility of these mechanisms for developing targeted, sex-informed interventions to reduce alcohol-related risk. Women may benefit from interventions targeting generalized anxiety symptoms to reduce stress-induced drinking, especially at higher levels of anxiety symptoms, whereas stimulation responses may inform risk processes in men. The amygdala and hippocampus may be potential neural targets for future mechanistic and interventional research. The divergence between generalized anxiety symptoms and state anxiety underscores the need for multi-level measurement to capture dynamic risk processes.

## Figures and Tables

**Table 1:** Linear and nonlinear growth curve models (GCMs) for men and women.

Variable		Linear GCM	Nonlinear GCM	Likelihood Ratio Test
**State Anxiety**	**χ^2^**	0.63 (1), *p* = 0.43	1.40 (2), *p* = 0.50	0.77 (1), *p* = 0.38
**Women**	***RMSEA***	<0.001	<0.001	
	***CFI***	1.00	1.00	
**State Anxiety**	**χ^2^**	0.19 (1), *p* = 0.66	1.18 (2), *p* = 0.55	0.99 (1), *p* = 0.32
**Men**	***RMSEA***	<0.001	<0.001	
	***CFI***	1.00	1.00	
**State Stimulation**	**χ^2^**	12.96 (1), *p* < 0.01	2.32 (2), *p* = 0.31	10.64 (1), *p* = 0.01
**Women**	***RMSEA***	0.51	0.06	
	***CFI***	0.85	0.10	
**State Stimulation**	**χ^2^**	12.89 (1), *p* < 0.01	0.73 (2), *p* = 0.70	12.16 (1), *p* < 0.01
**Men**	***RMSEA***	0.56	0.00	
	***CFI***	0.76	1.00	

Note: χ^2^ = chi-squared test statistic for model fit; numbers in parentheses are degrees of freedom for the chi-squared test statistic for model fit; *RMSEA* = root mean squared error of approximation; *CFI* = comparative fit index. The likelihood ratio test compares the nonlinear model to its corresponding linear model in terms of model fit. A non-significant result suggests that the linear and nonlinear models fit the data equally well; a significant result favors the nonlinear model.

**Table 2:** Sex differences in study variables.

Variable	Women		Men		*t*(df)	*p*	Cohen’s *d*
	*M*	*SD*	*M*	*SD*			
**Age**	30.83	10.71	33.42	10.45	−1.12(82)	0.13	−0.25
**CES-D**	11.40	9.82	10.54	8.03	0.42(78)	0.68	0.09
**GAD-7**	3.50	3.43	3.58	3.89	−0.10(82)	0.92	−0.02
**Neutral Alcohol-Seeking**	265.59	226.63	268.82	197.95	−0.07(82)	0.95	−0.02
**Stress Alcohol-Seeking**	265.24	200.90	284.34	177.43	−0.46(82)	0.65	−0.10
**Baseline State Anxiety**	21.28	26.20	9.95	20.57	2.17(82)	0.03	***0.48***
**Prime Start State Anxiety**	16.72	21.27	9.92	20.34	1.49(82)	0.14	0.33
**Prime End State Anxiety**	15.09	20.12	12.21	23.77	0.60(82)	0.55	0.13
**Baseline State Stimulation**	20.96	24.80	11.89	14.55	1.99(82)	0.05	***0.44***
**Prime Start State Stimulation**	37.48	27.23	33.16	23.68	0.77(82)	0.45	0.17
**Prime End State Stimulation**	35.72	26.25	36.37	24.75	−0.12(82)	0.91	−0.03
**TLFB Drinks Per Week**	23.40	34.78	21.27	18.69	0.34(82)	0.37	0.08
**TLFB Drinks Per Drinking Day**	5.59	4.94	5.54	3.40	0.05(82)	0.48	0.01
**TLFB Drinking Days Per Week**	3.60	1.78	3.84	1.66	−0.64(82)	0.26	−0.14

Note: CES-D = Center for Epidemiologic Studies Depression Scale; GAD-7 = Generalized Anxiety Disorder-7; TLFB = Timeline Follow-Back; *t* = Student’s *t* test; df = degrees of freedom; bold italics indicate *p* < 0.05.

**Table 3:** Correlations for study variables by sex.

Variable	1	2	3	4	5	6	7	8	9	10
**1. CES-D**	--	**0.60**	0.13	0.22	0.19	0.19	0.15	0.24	0.29	0.04
**2. GAD-7**	**0.61**	--	0.09	0.29	0.26	0.14	0.24	0.18	0.12	−0.12
**3. Neutral Seeking**	0.08	−0.02	--	**0.61**	0.07	0.19	0.1	**0.40**	0	0.12
**4. Stress Seeking**	−0.1	−0.19	**0.49**	--	−0.05	0.06	−0.12	**0.38**	−0.11	−0.08
**5. Baseline State Anxiety**	0.3	***0.37***	−0.17	***−0.36***	--	**0.8**	**0.65**	***0.31***	***0.32***	0.16
**6. Clamp Start State Anxiety**	0.06	0.2	−0.05	−0.31	**0.42**	--	**0.67**	***0.37***	0.24	0.14
**7. Clamp End State Anxiety**	0.21	0.11	0.17	−0.25	**0.42**	**0.65**	--	0.08	0.23	0.21
**8. Baseline Stimulation**	−0.08	−0.08	−0.09	−0.2	0.1	−0.14	−0.27	--	**0.42**	***0.35***
**9. Clamp Start Stimulation**	0.11	***0.35***	−0.16	−0.17	0.01	0.37	0.17	0.25	--	**0.89**
**10. Clamp End Stimulation**	0.15	0.29	−0.17	***−0.33***	0.22	0.39	***0.37***	***0.16***	***0.86***	--

Note: Men are found in the lower left triangle of the correlation matrix, with women in the upper right triangle. CES-D = Center for Epidemiologic Studies Depression Scale; GAD-7 = Generalized Anxiety Disorder-7; bold italics indicate *p* < 0.05; bold indicates *p* < 0.01.

**Table 4:** Moderating effect of sex on the relationship between anxiety symptoms and stress-induced alcohol-seeking.

Effect	Estimate	*SE*	95% CI		*p*
			*LL*	*UL*	
**Constant**	***86.15***	38.16	10.21	162.10	0.03
**GAD-7**	***13.90***	6.74	0.49	27.31	0.04
**Sex**	***95.19***	47.48	0.69	189.68	0.05
**GAD-7 × Sex**	***−22.01***	9.39	−40.70	−3.32	0.02
**Neutral Alcohol-Seeking**	**0.49**	0.08	0.33	0.65	<0.01

Note: GAD-7 = Generalized Anxiety Disorder-7; bold italics indicate *p* < 0.05; bold indicates *p* < 0.01; *SE* = Standard Error; CI = Confidence Interval; *LL*/*UL* = Lower/Upper Limit.

**Table 5:** Moderating effect of sex on the relationship between depression symptoms and stress-induced alcohol-seeking.

Effect	Estimate	*SE*	95% CI		*p*
			*LL*	*UL*	
**Constant**	***100.36***	41.16	18.36	182.36	0.02
**CES-D**	2.94	2.42	−1.89	7.76	0.23
**Sex**	75.84	57.03	−37.77	189.45	0.19
**CES-D × Sex**	−6.18	4.12	−14.40	2.03	0.14
**Neutral Alcohol-Seeking**	**0.51**	0.08	0.34	0.67	<0.01

Note: CES-D = Center for Epidemiologic Studies Depression Scale; bold italics indicate *p* < 0.05; bold indicates *p* < 0.01; *SE* = Standard Error; CI = Confidence Interval; *LL*/*UL* = Lower/Upper Limit.

**Table 6:** Moderated mediation model: state anxiety slope factor as mediator.

Outcome	Effect	Estimate	*SE*	*p*
**State Anxiety Change**				
	**GAD-7**	−0.00	0.01	0.47
	**Sex**	0.05	0.03	0.10
	**GAD-7 × Sex**	−0.00	0.01	0.63
	**Neutral Alcohol-Seeking**	0.01	0.01	0.11
**Stress Alcohol-Seeking**				
	**State Anxiety Change**	−8.36	4.73	0.08
	**Baseline State Anxiety**	**−3.06**	1.05	0.00
	**GAD-7**	***0.16***	0.07	0.01
	**Sex**	***1.03***	0.50	0.04
	**GAD-7 × Sex**	**−0.24**	0.09	0.01
	**Neutral Alcohol-Seeking**	**0.55**	0.08	<0.01

Note: GAD-7 = Generalized Anxiety Disorder-7; the slope factor captures linear change. Bold italics indicate *p* < 0.05; bold indicates *p* < 0.01; *SE* = Standard Error.

**Table 7:** Exploratory analysis: baseline state anxiety as mediator.

Outcome	Effect	Estimate	*SE*	*p*
**Baseline State Anxiety**				
	**Constant**	15.13	5.71	0.10
	**GAD-7**	2.00^†^	1.01	0.05
	**Sex**	−11.28	7.11	0.12
	**GAD-7 × Sex**	−0.06	1.41	0.97
	**Neutral Alcohol-Seeking**	−0.00	0.01	0.79
**Stress Alcohol-Seeking**				
	**Constant**	**108.33**	39.09	0.01
	**Baseline State Anxiety**	**−1.47**	0.74	0.00
	**GAD-7**	***16.83***	6.78	0.02
	**Sex**	78.65	47.35	0.10
	**GAD-7 × Sex**	***−22.09***	9.22	0.02
	**Neutral Alcohol-Seeking**	**0.49**	0.08	<0.01

Note: GAD-7 = Generalized Anxiety Disorder-7; bold italics indicate *p* < 0.05; bold indicates *p* < 0.01; *SE* = Standard Error; ^†^*p* < 0.1.

**Table 8:** Moderated mediation model: state stimulation shape factor as mediator.

Outcome	Effect	Estimate	*SE*	*p*
**Stimulation Growth Curve**				
	**GAD-7**	−0.01	0.01	0.25
	**Sex**	−0.05	0.07	0.51
	**GAD-7 × Sex**	***0.03***	0.02	0.02
	**Neutral Seeking**	−0.01	0.01	0.26
**Stress Alcohol-Seeking**				
	**State Stimulation Growth Curve**	−1.56	0.86	0.07
	**Baseline State Stimulation**	−0.27	1.04	0.80
	**GAD-7**	0.12	0.07	0.06
	**Sex**	0.87	0.46	0.06
	**GAD-7 × Sex**	−0.17	0.09	0.07
	**Neutral Alcohol-Seeking**	**0.46**	0.08	<0.01

Note: GAD-7 = Generalized Anxiety Disorder-7; the shape factor captures nonlinear change. Bold italics indicate *p* < 0.05; bold indicates *p* < 0.01; *SE* = Standard Error.

## Data Availability

The raw data supporting the conclusions of this article will be made available by the authors upon request.

## Supplementary Materials

The following supporting information can be downloaded at https://www.mdpi.com/article/10.3390/bs16020311/s1: Table S1: Sample characteristics comparisons across imaged and non-imaged samples; Table S2: Descriptive statistics and correlations for study variables; Table S3: Moderated mediation model: depression as predictor, state anxiety slope factor as mediator; Table S4: Moderated medication model: depression as predictor, state stimulation shape factor as mediator.
