# Supplementary material for: Sex-Based Mechanisms of Stress-Induced Alcohol-Seeking
Source: Behav Sci (Basel). 2026 Feb 23;16(2):311. doi: 10.3390/bs16020311 (PMC12938385; doi:10.3390/bs16020311)
Supplement: Supplementary file 1 [file behavsci-16-00311-s001.zip › behavsci-3953644-supplementary.pdf]

behavioral sciences

MDPI

Supplemental Materials

Table S1. Sample characteristics comparisons across imaged and non-imaged samples.

Variable	Full Sample (n = 84)	Imaged (n = 54)	Non-Imaged (n = 30)	Comparison
**Sex**	46 female<br>38 male	31 female<br>23 male	15 female<br>15 male	$\chi^2 = 0.43$, $p = 0.65$
**Race**	1 American Indian<br>2 Asian<br>27 Black/African American<br>3 More than one race<br>2 Unknown<br>49 White	0 American Indian<br>2 Asian<br>16 Black/African American<br>2 More than one race<br>1 Unknown<br>33 White	1 American Indian<br>0 Asian<br>11 Black/African American<br>1 More than one race<br>1 Unknown<br>16 White	$\chi^2 = 3.59$, $p = 0.61$
**Ethnicity**	74 Not Hispanic/ Latino<br>10 Hispanic/Latino	47 Not Hispanic/ Latino<br>7 Hispanic/Latino	27 Not Hispanic/ Latino<br>3 Hispanic/Latino	$\chi^2 = 0.16$, $p = 1.00$
**Age**	Mean = 32.0<br>(SD = 10.6),<br>Range 21–55	Mean = 32.28<br>(SD = 10.05),<br>Range 21–55	Mean = 31.50<br>(SD = 11.70),<br>Range 21–54	$t(82) = -0.32$, $p = 0.75$
**CES-D**	Mean = 11.02<br>(SD = 9.03),<br>Range 0–48	Mean = 11.19<br>(SD = 8.99),<br>Range 0–48	Mean = 10.71<br>(SD = 9.27),<br>Range 0–35	$t(78) = -0.22$, $p = 0.82$
**GAD-7**	Mean = 3.54<br>(SD = 3.62),<br>Range 0–17	Mean = 3.43<br>(SD = 3.44),<br>Range 0–17	Mean = 3.73<br>(SD = 3.99),<br>Range 0–15	$t(82) = 0.37$, $p = 0.71$
**Neutral Alcohol-Seeking**	Mean = 267.10<br>(SD = 212.9),<br>Range 1–738	Mean = 252.0<br>(SD = 217.52),<br>Range 1–707	Mean = 294.13<br>(SD = 205.07),<br>Range 4–738	$t(82) = -0.86$, $p = 0.39$
**Stress Alcohol-Seeking**	Mean = 273.90<br>(SD = 189.8),<br>Range 1–708	Mean = 275.11<br>(SD = 194.78),<br>Range 1–708	Mean = 271.67<br>(SD = 183.61),<br>Range 30–623	$t(82) = -0.08$, $p = 0.94$
**TLFB Drinks Per Week**	Mean = 22.40<br>(SD = 28.51),<br>Range 3.2–182	Mean = 21.53<br>(SD = 26.91),<br>Range 3.4–182	Mean = 24.07<br>(SD = 31.58),<br>Range 3.2–126.8	$t(82) = 0.39$, $p = 0.35$
**TLFB Drinks Per Drinking Day**	Mean = 5.57<br>(SD = 4.29),<br>Range 1.7–26	Mean = 5.32<br>(SD = 4.11),<br>Range 1.7–26	Mean = 6.01<br>(SD = 4.63),<br>Range 1.78–19.21	$t(82) = 0.70$, $p = 0.24$
**TLFB Drinking Days Per Week**	Mean = 3.70<br>(SD = 1.72),<br>Range 0.6–7	Mean = 3.84<br>(SD = 1.70),<br>Range 0.6–7	Mean = 3.46<br>(SD = 1.76),<br>Range 1–7	$t(82) = -0.97$, $p = 0.17$

Note: CES-D = Center for Epidemiologic Studies Depression Scale; GAD-7 = Generalized Anxiety Disorder-7; TLFB = Timeline Follow-Back; SD = standard deviation; χ^2 = chi-squared test; t = Student's t test; numbers in parentheses are degrees of freedom.

Table S2. Descriptive statistics and correlations for study variables.

Variable	n	M	SD	1	2	3	4	5	6	7	8	9	10
**1. CES-D**	80	11.03	9.03	--									
**2. GAD-7**	84	3.54	3.62	**0.60**	--								
**3. Neutral Alcohol-Seeking**	84	267.05	212.88	0.11	0.04	--							
**4. Stress Alcohol-Seeking**	84	273.88	189.76	0.10	0.07	**0.57**	--						

**5. Baseline State Anxiety**	84	16.15	24.35	***0.24***	**0.29**	−0.02	−0.17	--					
**6. Prime Start State Anxiety**	84	13.64	21.01	0.15	0.16	0.09	−0.10	**0.66**	--				
**7. Prime End State Anxiety**	84	13.79	21.75	0.17	0.17	0.13	−0.18	**0.54**	**0.66**	--			
**8. Baseline State Stimulation**	84	16.86	21.18	0.16	0.08	***0.24***	0.19	**0.29**	***0.23***	−0.03	--		
**9. Prime Start State Stimulation**	84	35.52	25.63	***0.23***	***0.22***	−0.06	−0.14	***0.22***	**0.31**	0.20	**0.37**	--	
**10. Prime End State Stimulation**	84	36.01	25.43	0.08	0.07	0.01	−0.18	0.17	***0.24***	**0.28**	***0.28***	**0.87**	--

Note: CES-D = Center for Epidemiologic Studies Depression Scale; GAD-7 = Generalized Anxiety Disorder-7; bold italics indicate $p < 0.05$; bold indicates $p < 0.01$.

Table S3. Moderated mediation model: depression as predictor, state anxiety slope factor as mediator.

Outcome	Effect	Estimate	*SE*	*p*
**State Anxiety Slope**				
	**CES-D**	−0.00	0.00	0.47
**Factor**	**Sex**	0.04	0.04	0.30
	**CES-D × Sex**	0.00	0.00	0.99
	**Neutral Alcohol-Seeking**	0.01	0.01	0.11
**Stress Alcohol-Seeking**				
	**State Anxiety Change**	−7.83	4.67	0.09
	**Baseline State Anxiety**	**−2.89**	1.07	0.01
	**CES-D**	0.03	0.02	0.16
	**Sex**	0.82	0.57	0.15
	**CES-D × Sex**	−0.06	0.04	0.15
	**Neutral Alcohol-Seeking**	**0.55**	0.09	<0.01

Note: CES-D = Center for Epidemiologic Studies Depression Scale; the slope factor captures linear change; bold indicates $p < 0.01$; *SE* = Standard Error.

Table S4. Moderated mediation model: depression as predictor, state stimulation shape factor as mediator.

Outcome	Effect	Estimate	*SE*	*p*
**Stimulation Shape Factor**				
	**CES-D**	−0.00	0.00	0.77
	**Sex**	0.00	0.09	0.97
	**CES-D × Sex**	0.01	0.01	0.31
	**Neutral Alcohol-Seeking**	−0.01	0.01	0.23
**Stress Alcohol-Seeking**				
	**Stimulation Growth Curve**	***−1.80***	0.84	0.03
	**Baseline Stimulation**	−0.37	1.08	0.74
	**CES-D**	0.03	0.02	0.21
	**Sex**	0.86	0.53	0.11
	**CES-D × Sex**	−0.05	0.04	0.18
	**Neutral Alcohol-Seeking**	**0.46**	0.08	<0.01

Note: CES-D = Center for Epidemiologic Studies Depression Scale; the shape factor captures nonlinear change; bold italics indicate $p < 0.05$; bold indicates $p < 0.01$;. *SE* = Standard Error.
